# Martial arts training and aggressive behavior in children and adolescents: a systematic review of longitudinal evidence and psychological processes

**DOI:** 10.3389/fpubh.2026.1838396

**Published:** 2026-07-17

**Authors:** Lei Deng, Xiujie Ma, Qingyuan Luo

**Affiliations:** 1 School of Martial Arts, Chengdu Sport University, Chengdu, China; 2 Hongyi College, Chengdu Technological University, Chengdu, China; 3 Chinese Guoshu Academy, Chengdu Sport University, Chengdu, China

**Keywords:** aggressive behavior, children and adolescents, martial arts, self-control, social adaptation, systematic review

## Abstract

Aggressive behavior in childhood and adolescence represents a persistent developmental and public health concern, with important implications for psychosocial adjustment and long-term wellbeing. Martial arts training, which typically integrates structured physical activity with rule-based interaction, discipline, and social learning, has increasingly been proposed as a potential intervention context for shaping behavioral and emotional development. However, longitudinal evidence regarding its relationship with youth aggression remains fragmented and heterogeneous. This systematic review synthesized longitudinal intervention studies examining the relationship between martial arts training and changes in aggressive behavior among children and adolescents. A comprehensive search of international and Chinese databases identified 11 eligible studies, including two randomized controlled trials and nine quasi-experimental intervention studies. Methodological quality was assessed using the RoB 2 tool for randomized trials and the JBI Critical Appraisal Checklist for quasi-experimental studies. Overall, the evidence does not indicate a consistent directional effect of martial arts interventions on aggressive behavior. Instead, findings varied substantially across martial arts disciplines, intervention structures, and research contexts. Across studies, psychological variables relevant to potential psychological processes were mainly represented in two domains: (1) self-regulation-related processes, including self-control and emotion-related indicators; and (2) socialization-related constructs, including self-concept, peer-related indicators, and broader psychosocial functioning. However, considerable variation existed in the conceptualization and measurement of these variables, limiting direct comparability across studies and preventing firm conclusions about causal mechanisms. Taken together, martial arts training should not be interpreted as having a uniform aggression-reducing effect among children and adolescents. Its potential value appears conditional on educational goals, pedagogical guidance, and training context. Future research should clarify when, for whom, and through what pathways martial arts training may influence aggression-related outcomes.

## 1 Introduction

### 1.1 Child and adolescent aggression as a critical public health and developmental issue

Aggressive behavior refers to actions intended to harm others or damage their property, which may manifest through physical aggression, verbal hostility, or indirect forms such as psychological manipulation (1). Among children and adolescents, aggression commonly appears in forms such as school bullying, peer violence, and relational aggression. These behaviors represent persistent challenges in school contexts and are associated with a range of negative consequences for psychological wellbeing, social adjustment, and the overall safety of educational environments.

A substantial body of research indicates that aggressive behavior in children and adolescents is closely associated with emotional distress, interpersonal conflict, and difficulties in academic and social functioning. Importantly, aggression during this developmental period often demonstrates continuity over time (2). Longitudinal studies suggest that higher levels of aggression in children and adolescents are associated with increased risks of depression, anxiety, substance misuse, and antisocial behavior later in life (3). Moreover, both individuals who engage in aggressive behavior and those who experience prolonged exposure to bullying or violent environments appear to face elevated risks of long-term mental health problems and impaired social functioning (4).

From a developmental psychology perspective, childhood and adolescence constitute critical periods for the emergence and consolidation of aggressive behavior. Neurocognitive research suggests that executive functions, self-control, and emotion regulation capacities are still developing during these stages, while emotional reactivity and sensitivity to social cues may remain relatively high. This developmental imbalance can increase the likelihood of impulsive or aggressive responses when individuals encounter interpersonal conflict or provocation (5). At the same time, peer interactions play a central role in shaping behavioral norms and social learning processes among children and adolescents. Peer influence, observational learning, and social feedback within group contexts may amplify behavioral responses and contribute to the emergence and maintenance of aggressive behavior (6).

From a public health perspective, aggression among children and adolescents should not be viewed solely as an individual behavioral problem but rather as a widespread social risk behavior with significant societal implications. According to a report by the United Nations Educational, Scientific and Cultural Organization, approximately one-third of adolescents worldwide have experienced some form of school bullying (7), indicating that this problem is widespread across diverse cultural and educational contexts. The World Health Organization has also recognized violence—including aggressive behavior among children and adolescents—as a major global public health concern (8). Aggressive behavior not only increases the risk of physical injury and psychological trauma but may also disrupt peer relationships, undermine school climates, and generate broader social consequences.

Taken together, accumulating evidence suggests aggression in childhood and adolescence is not merely a transient behavioral problem but a complex developmental phenomenon embedded within psychological maturation and socialization processes. Consequently, identifying effective strategies to intervene during childhood and adolescence has become a shared priority across psychology, education, and public health. From an intervention perspective, relying solely on external behavioral control is unlikely to produce sustained improvements. Greater attention should therefore be directed toward the psychological developmental processes of children and adolescents, including self-regulation, emotional management, social cognition, and the internalization of social norms. This perspective provides an important theoretical foundation for exploring intervention approaches that are developmentally appropriate and capable of supporting long-term behavioral change.

### 1.2 Martial arts–based interventions for aggression in children and adolescents

In recent years, martial arts training has increasingly been introduced into research on interventions for aggressive behavior among children and adolescents and has been considered a form of intervention that integrates physical activity with psychological regulation. Compared with general physical activities, martial arts training is typically embedded within more structured rule systems and social contexts, such as etiquette norms, disciplinary requirements, peer interaction, and instructor–student relationships. These characteristics have led scholars to regard martial arts training as a potentially comprehensive training approach that may simultaneously influence physical, psychological, and social behavioral dimensions in aggression intervention. Consequently, increasing attention has been devoted in recent years to examining the role of martial arts training in interventions targeting aggressive behavior among children and adolescents and to exploring the psychological processes that may accompany martial arts training. Early empirical studies examining the relationship between martial arts practice and aggression have also produced mixed findings. For example, some early investigations reported lower levels of hostility among martial arts practitioners compared with non-practitioners (9, 10).

Existing empirical studies suggest that, under certain conditions, martial arts training may contribute to reductions in physical aggression (11, 12), verbal aggression (13, 14), and indirect forms of aggression (15) among children and adolescents. For example, Fung and Lee (16) reported that adolescents participating in martial arts training demonstrated significantly lower levels of proactive aggression, an effect that may be closely related to the emphasis on rule awareness, behavioral regulation, and moral norms within training environments. Similarly, Greco et al. (17) found that adolescents receiving martial arts training exhibited greater psychological adaptability when facing bullying or conflict situations and were more likely to adopt non-violent strategies for conflict resolution.

In addition to intervention-based evidence, cross-sectional investigations have also explored the relationship between martial arts participation and aggression-related traits, although their findings remain inconsistent across contexts (18–21).

However, existing findings remain inconsistent, and caution is warranted when interpreting the effects of martial arts training on aggressive behavior. On the one hand, substantial variations exist across studies in terms of research design, sample characteristics, training content and instructional orientation, intervention duration, and measurement of aggression, which may contribute to considerable heterogeneity in both the direction and magnitude of observed effects. On the other hand, previous review studies have noted that although overall trends provide some support for the positive role of martial arts training in psychological adaptation and behavioral regulation, its effectiveness in reducing aggression has not been consistently confirmed across all studies, and most research has primarily focused on intervention outcomes rather than systematically examining the underlying psychological processes (22, 23). It is also noteworthy that in training environments emphasizing competitiveness, performance outcomes, or physical dominance, certain combat or strength-oriented sports may not necessarily reduce aggression and may even be associated with higher levels of aggressive or antisocial behavior. This observation further suggests that the effects of martial arts training are highly dependent on specific training content, instructional orientation, and the broader sociocultural context in which training occurs (24), rather than being a simple consequence of “martial arts training” itself.

Taken together, although existing research provides partial support for the potential value of martial arts training as an intervention approach for aggressive behavior among children and adolescents, its effects and associated psychological changes appear to be complex and heterogeneous. Compared with intervention outcomes themselves, the psychological processes potentially involved in different training orientations of martial arts remain relatively fragmented and have yet to be systematically integrated within a coherent theoretical framework. Therefore, a systematic review from the perspective of psychological processes is needed to provide a more comprehensive understanding of the characteristics and current state of research on martial arts training in interventions targeting aggressive behavior among children and adolescents.

### 1.3 Psychological perspectives on martial arts training and aggression

Previous research has generally suggested that the potential value of martial arts training in addressing aggressive behavior among children and adolescents lies not only in observable behavioral changes but also in its influence on psychological functioning and behavioral regulation processes (25). However, empirical findings remain inconsistent, and most studies have primarily focused on changes in aggression levels before and after intervention while providing limited systematic examination of the psychological processes that may occur during the intervention period (22). As a result, evaluating martial arts interventions solely in terms of whether aggression decreases may not fully capture how such training operates across different research contexts.

Research in developmental psychology and behavioral science commonly conceptualizes aggressive behavior as a complex developmental outcome embedded within individuals' social cognition, emotional responses, and behavioral regulation processes (26). Among these processes, emotion-related self-regulation abilities—particularly the development of effortful control—are widely considered a critical psychological foundation for adaptive functioning in children and adolescents, and their development is shaped by dynamic interactions between genetic and environmental factors (27). Understanding how martial arts training may influence these key psychological processes is therefore essential for explaining inconsistencies in existing findings and for improving the precision of intervention strategies.

From a theoretical perspective, cognitive–behavioral theory provides an important framework for interpreting how martial arts training may influence aggressive behavior. This perspective emphasizes that behavioral change depends on the interaction among cognitive processing patterns, emotional regulation capacities, and behavioral control strategies rather than relying solely on external behavioral suppression (28). Within this framework, martial arts training may indirectly influence individuals' responses to conflict or provocation by shaping self-regulation abilities, social cognitive structures, and value orientations.

More specifically, previous studies have examined the potential effects of martial arts training from multiple psychological dimensions, including emotion regulation and impulse control (29), self-efficacy and psychological resilience (30–32), self-esteem and subjective wellbeing (33–35), peer motivational climate (36), and moral beliefs (37). Nevertheless, most of these studies have focused on single or limited dimensions, and considerable variation exists in conceptual definitions, measurement approaches, and interpretive perspectives, resulting in a lack of systematic integration across findings.

In light of these considerations, a systematic review of the psychological processes associated with martial arts interventions for aggressive behavior in children and adolescents is warranted. Integrating the diverse psychological dimensions examined in previous studies may help clarify the patterns underlying existing findings and provide a more coherent theoretical basis for understanding how martial arts training relates to aggression-related outcomes.

### 1.4 Research gaps and the aims of the present systematic review

Although existing studies provide some support for the positive influence of martial arts training on aggressive behavior and related indicators of psychological adaptation among children and adolescents, several important limitations remain that constrain the systematic integration of findings and the advancement of theory. First, much of the current research has primarily focused on intervention outcomes—namely, whether martial arts training is effective—while offering limited systematic examination of the psychological changes and processes that may occur during the intervention period. Second, martial arts training itself is characterized by substantial heterogeneity in terms of program types, instructional goals, and implementation contexts. As a result, different studies often adopt varying definitions of what constitutes “martial arts training.” However, relatively few studies have systematically compared the psychological characteristics emphasized across different training orientations from the perspective of psychological processes, which makes it difficult to directly interpret and integrate findings across studies.

In light of these issues, a systematic synthesis of research on martial arts interventions targeting aggressive behavior in children and adolescents from the perspective of psychological processes is warranted. The present study therefore adopts a systematic review approach to integrate relevant empirical evidence from both international and domestic literature. Through rigorous literature screening and quality assessment, this review aims to provide a comprehensive overview of the types of psychological processes examined in existing studies and their corresponding research characteristics.

To address these gaps, the present study conducted a systematic review of longitudinal intervention studies examining martial arts training and aggressive behavior in children and adolescents. The review was guided by three research questions:

(1) What empirical evidence exists regarding the effects of martial arts training on aggressive behavior and related outcomes among children and adolescents?
(2) What psychological variables have been examined in previous intervention studies to interpret the relationship between martial arts training and aggression-related outcomes?
(3) What patterns can be identified in how psychological processes are represented across different martial arts training contexts and research designs?

### 1.5 Conceptual scope and terminological clarifications

Within the international academic context, the term “martial arts” does not refer exclusively to Chinese martial arts but instead represents a broader category encompassing a variety of combat-based and mind–body training practices that originate from East Asian and related cultural traditions (38). These include, but are not limited to, Chinese traditional martial arts, taekwondo, judo, karate, and sanda. Accordingly, the term “martial arts” is used throughout this review to correspond to the concept of martial arts in international literature and to refer collectively to these diverse training practices rather than to any single technical system.

## 2 Methods

This systematic review was registered in the International Prospective Register of Systematic Reviews (PROSPEROD; registration number: CRD^*************^). The review was retrospectively registered on 30 January 2026; therefore, the registration should be interpreted as a transparency measure rather than as evidence of prospective protocol registration.

To ensure transparency and methodological rigor, the review was conducted in accordance with the Preferred Reporting Items for Systematic Reviews and Meta-Analyses (PRISMA 2020) guidelines. The review process included systematic literature searching, pre-defined eligibility criteria, structured study selection, standardized data extraction, psychological variable identification and classification, methodological quality assessment, and narrative synthesis. The identification and classification process was documented using a structured codebook (Supplementary material A4), which specified the variables to be extracted and provided guidance for consistent data recording, while allowing the higher-order psychological categories to be refined inductively during analysis.

In addition to synthesizing aggression-related outcomes, the review included an analytical focus on psychological variables reported in relation to aggression outcomes within the included intervention studies.

For the purpose of systematic data management and clear presentation of the findings, the 11 studies included in this review were assigned unique identification numbers according to publication year, from most recent to earliest. Each study was labeled sequentially (e.g., S1, S2) and these identifiers were used consistently throughout the tables and Supplementary materials to ensure accurate referencing and traceability of the included studies.

### 2.1 Literature sources and search strategy

Electronic literature searches were conducted in Web of Science, PubMed, ScienceDirect, and Google Scholar to identify relevant English-language studies. Additional records were identified through backward citation tracking and supplementary searches in Google Scholar. Chinese-language studies were identified primarily through the China National Knowledge Infrastructure (CNKI) and Wanfang databases. All databases were searched from database inception to January 2025. Accordingly, the findings of this review should be interpreted as a synthesis of evidence available up to that search date. The search aimed to identify empirical studies examining the effects of martial arts training on aggressive behavior in children and adolescents.

Based on the primary research objective, the search strategy was developed around three core conceptual domains: martial arts training, aggressive behavior or related externalizing outcomes, and the target population. Search terms were applied to title, abstract, or topic fields depending on the indexing rules of each database. Boolean operators (AND, OR) were used to combine keywords.

The search strategy was designed to identify studies in which aggression-related outcomes were reported in the context of martial arts training. Psychological variables were not included as an independent search domain because preliminary scoping searches indicated that many relevant studies discussed psychological processes in relation to aggression without using psychological constructs as primary keywords, titles, or eligibility-defining outcomes. Including broad psychological terms in the main search strategy could therefore have reduced search sensitivity and increased the risk of missing eligible studies that reported aggression-related outcomes. Accordingly, psychological variables were examined during data extraction and synthesis when they were reported alongside aggression-related outcomes.

Search strategies were adapted to the syntax and interface of each database while maintaining a consistent conceptual framework. The complete search strategies for each database are provided in Supplementary material A1 (S1).

English search terms included:

(1) Martial arts categories: martial arts, kung fu, wushu, Tai Chi, taiji, taekwondo, karate, judo, aikido, MMA, mixed martial arts;
(2) Aggression-related terms: aggression, aggressive, aggressiveness, hostility, violence, anger, bully, bullying;
(3) Population terms: adolescents, youth, students, teenagers, children, boys, girls.

Chinese search terms included:

(1) Martial arts categories: 武术, 散打, 太极, 跆拳道, 空手道, 柔道, 格斗;
(2) Aggression-related terms: 攻击, 攻击性, 攻击行为, 校园欺凌, 校园霸凌;
(3) Population terms: 青少年, 儿童, 学生, 少年.

### 2.2 Eligibility criteria

#### 2.2.1 Inclusion criteria

Studies were included if they met the following criteria:

(1) Participants were children or adolescents (typically aged 18 years or younger).
(2) The study examined martial arts training as the primary intervention or research focus, including disciplines such as taekwondo, karate, tai chi, sanda, or traditional Chinese martial arts, with structured instruction or sustained practice.
(3) The study reported aggressive behavior or related externalizing outcomes, such as physical aggression, verbal aggression, hostility, anger, or school bullying.
(4) The study adopted a longitudinal design, including randomized controlled trials, quasi-experimental studies, or pre–post intervention designs that allowed comparisons across time or between groups.
(5) The study employed quantitative methods and used standardized or structured instruments to assess aggressive behavior.

#### 2.2.2 Exclusion criteria

Studies were excluded if they met any of the following conditions:

(1) Participants were adults (including university students) or the age group was not clearly identified as children or adolescents.
(2) Aggressive behavior or related externalizing outcomes were not reported.
(3) Martial arts training was not the primary intervention.
(4) The study used a cross-sectional design without longitudinal comparison.
(5) The study was qualitative, case-based, or lacked quantitative data.
(6) Conference abstracts, review articles, commentaries, dissertations, or unpublished studies.
(7) Publications not written in English or Chinese.

### 2.3 Study selection and data extraction

To ensure the rigor and consistency of the study selection process, two reviewers independently screened all retrieved records according to the pre-defined eligibility criteria. Any disagreements that arose during the screening process were resolved through discussion until consensus was reached. After study selection, data from the included studies were extracted using a structured data extraction form (Supplementary material A3). Data extraction was conducted by one reviewer and independently checked by a second reviewer to ensure accuracy and consistency. Any discrepancies in extracted information were resolved through discussion and consensus. Extracted information included study characteristics, participant information, intervention features, aggression-related outcomes, and psychological variables reported alongside aggression-related outcomes.

Using the search strategy described above, systematic searches were conducted across both English- and Chinese-language databases. A total of 834 records were initially identified, including 546 English-language studies and 288 Chinese-language studies. In addition, 58 records were identified through supplementary searches using Google Scholar and backward citation tracking.

All database records were imported into Zotero reference management software for organization and screening. After removing 274 duplicate records, attempts were made to retrieve full texts through available institutional subscriptions, publisher websites, DOI links, Google Scholar, CNKI, Wanfang, and reference tracing. A total of 172 records could not be retrieved in full text after these attempts and were therefore excluded before eligibility assessment. The remaining 388 records proceeded to title and abstract screening.

During the first screening stage, 320 records were excluded for the following reasons: topic irrelevance (*n* = 208) and non-original publications, including reviews, dissertations, and conference abstracts (*n* = 112). As a result, 68 reports proceeded to the full-text eligibility assessment stage.

In the second screening stage, a further 60 reports were excluded for the following reasons: not related to violence or aggression (*n* = 17), non-adolescent samples (*n* = 13), non-empirical studies (*n* = 21), and cross-sectional designs (*n* = 9). After this stage, eight studies from database sources met the inclusion criteria.

**Figure 1:**
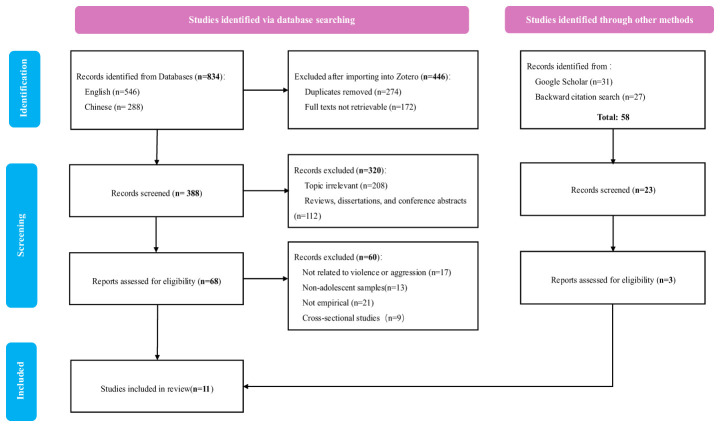
PRISMA 2020 flow diagram of the literature selection process.

From the additional sources, 58 records were identified. After preliminary screening, 23 records entered the title and abstract screening stage, and three studies proceeded to full-text assessment. After combining the database and additional sources, a total of 11 studies were ultimately included in the systematic review. The detailed study selection process is illustrated in the PRISMA 2020 flow diagram (Figure 1).

### 2.4 Quality appraisal

To ensure the methodological rigor of the present systematic review, a stratified risk-of-bias assessment strategy was adopted according to the research design of the included studies.

For randomized controlled trials, the Risk of Bias 2 (RoB 2) tool recommended by the Cochrane Collaboration was used. This tool evaluates potential bias across five domains: the randomization process, deviations from intended interventions, missing outcome data, measurement of the outcome, and selection of the reported result. Based on the judgments across these domains, each study is assigned an overall risk-of-bias level (low risk, some concerns, or high risk).

For non-randomized intervention studies and single-group pre–post studies, the Joanna Briggs Institute (JBI) Critical Appraisal Checklist for Quasi-Experimental Studies was applied. This tool evaluates methodological quality across several domains, including temporal sequence of intervention and outcome, comparability of participants, control of potential confounding factors, consistency of intervention implementation, outcome measurement methods, reliability of outcome assessment, completeness of follow-up, and appropriateness of statistical analysis.

Risk-of-bias assessment was conducted independently by two reviewers. Disagreements were resolved through discussion until consensus was reached. The results of the risk-of-bias assessments were reported by domain in the Results section and were used to identify potential methodological limitations in the included studies, thereby informing the interpretation and synthesis of the evidence.

### 2.5 Psychological variable identification and classification

During the full-text review and data extraction process, all included studies were examined to identify psychological variables reported in relation to aggressive behavior outcomes. In addition to recording aggression-related outcome measures, psychological variables were extracted when they were used as outcome indicators, explanatory variables, or interpretive constructs related to the relationship between martial arts training and aggressive behavior. The identification and classification of psychological variables followed the same extraction-and-checking procedure described above, with classification decisions checked by a second reviewer and discrepancies resolved through discussion and consensus.

The review incorporated an analytical focus on psychological processes related to aggression outcomes. However, no fixed higher-order classification scheme was imposed before data extraction. Instead, psychological variables were first identified from the included studies and were then organized according to their functional characteristics and conceptual similarities.

Through a comparative examination of outcome indicators, psychological variables, and their corresponding interpretations across the included studies, several recurring psychological constructs were identified. Although the included studies differed in terms of intervention formats, research designs, and measurement instruments, many referred to constructs associated with behavioral regulation, emotional management, self-evaluation, peer interaction, and social adaptation.

Based on these patterns, the psychological variables were broadly categorized into two groups: (1) self-regulation-related variables, including constructs associated with impulse control, self-control, and emotion regulation; and (2) socialization-related variables, including constructs related to normative awareness, value internalization, peer relationships, self-concept, and social adaptation.

This classification approach was used to provide a structured synthesis of the psychological variables reported in included intervention studies, while allowing the categories to remain grounded in the concepts and measures represented in the included literature.

### 2.6 Data synthesis strategy

Given the small number of eligible studies and the substantial heterogeneity across study designs, martial arts disciplines, intervention duration and intensity, participant characteristics, outcome constructs, and measurement instruments, a quantitative meta-analysis was not conducted. Instead, a structured narrative synthesis approach was adopted.

The synthesis was organized in four steps. First, the characteristics of included studies were tabulated, including participant information, intervention features, research design, measurement instruments, and outcome domains. Second, aggression-related findings were summarized according to martial arts discipline and direction of reported change, including decrease, increase, no significant change, or mixed findings. Third, psychological variables reported alongside aggression-related outcomes were identified from the extracted outcome variables and narratively grouped into self-regulation-related and socialization-related domains during synthesis. Fourth, patterns of heterogeneity were examined across study design, intervention structure, outcome operationalization, participant characteristics, and cultural or educational context.

## 3 Results

### 3.1 Study selection

Based on the pre-defined search strategy and eligibility criteria, a total of 11 studies were ultimately included in the present systematic review. The overall characteristics of the included studies—including author information, publication year, country or region, sample characteristics, intervention parameters, research design, measurement tools, types of martial arts intervention, and primary outcome measures—are summarized in Table 1.

**Table 1:** Characteristics of included studies.

Study ID	References	Country/ region	*N*	Age/grade^1^	Intervention dose (total duration, session frequency, session length)	Gender male (%)	Research design	Measurement tools^2^	Intervention (martial arts discipline)	Outcome measures
S1	Ortiz-Franco et al., (39)	Spain	139	14–18	12 weeks; 2 sessions/week; 60 min/session	50.3	Non-randomized	SSRI; VBSS	Judo	overt aggression; relational aggression; emotional perception; self-emotional management; hetero-emotional management; emotional use
S2	Lafuente et al., (44)	Spain	82	9–12	6 months; 2–3 sessions/week; 60 min/session	63.4	Non-randomized	STAXI-NA	Judo, Karate	trait anger; anger expression; anger control
S3	Moore et al., (14)	Australia	243	12–14	10 weeks; 1 session/week; 60 min/session	56	RCT	SDQ	Taekwondo	aggressive behavior; emotional difficulties; behavior difficulties; total difficulties
S4	Lindell-Postigo et al., (29)	Spain	139	11–16	2 months; 8 sessions total; session length NR (school-based PE)	50.4	Non-randomized	SCF-5; PMCSQ-2; SSRI; SVS	Judo	aggressive behavior; emotional intelligence; self-concept; motivational climate
S5	Harwood-Gross et al., (45)	Israel	39	14–18	6 months; 2 sessions/week; 50 min/session	100	Non-randomized	CANTAB; RSES; AS	Martial arts (Karate, Ju-jitsu, Judo)	executive functions; aggression; self-esteem; hormonal reactivity
S6	Fung and Lee, (16)	Hong Kong, China	298	Grades 2–5	10 weeks; 1 session/week; 90 min/session	77.8	RCT	RPQ; CBCL-YSR	Chinese martial arts	reactive aggression; proactive aggression; general aggression; delinquent behavior; anxious/de-pressed; attention problem
S7	Twemlow et al., (46)	USA	238	Grades 3–5	3 sessions total; 45 min/session; booster format	57.9	Non-randomized	VO; VS; AL	Traditional martial arts (unspecified)	aggression; helpful bystander behavior; victimization
S8	Reynes and Lorant, (42)	France	43	8–10	2 years; frequency NR; session length NR	100	Non-randomized	BPAQ	Judo, Karate	physical aggression; verbal aggression; anger; hostility
S9	Reynes and Lorant, (43)	France	55	8	1 years; 2 sessions/week; 90 min/session	100	Non-randomized	BPAQ	Judo	physical aggression; verbal aggression; anger; hostility
S10	Delva-Tauiliili, (41)	USA	42	9–12	2.5 weeks; frequency NR; session length NR	100	Non-randomized	TSCRS; ABS	Aikido	aggression; self-control
S11	Trulson, (40)	USA	34	13–17	6 months; 3 sessions/week; 60 min/session	100	Non-randomized	MMPI; PFT; JPI	Taekwondo, Modern martial arts	aggressiveness; delinquency-related personality traits; anxiety; self-esteem

^1^Age and grade information are reported as described in the original studies. Values represent age ranges in years unless reported as school grade ranges (e.g., Grades 2–5) when exact age data were unavailable.

^2^SSRI, Schutte Self-Report Inventory; VBSS, Violent Behavior at School Scale; STAXI-NA, State-Trait Anger Expression Inventory for children and adolescents; SDQ, Strengths and Difficulties Questionnaire; SCF-5, self-concept form-5; PMCSQ-2, Perceived Motivational Climate in Sport Questionnaire-2; SVS, School Violence Scale; CANTAB, Cambridge neuropsychological test automated battery; RSES, Rosenberg Self Esteem Scale; AS, Aggression Scale; RPQ, Reactive-Proactive Aggression Questionnaire; CBCL-YSR, child behavioral checklist–youth self-report; VO, Victimization of Others Scale; VS, Victimization of Self Scale; AL, Aggression is Legitimate Scale; BPAQ, Buss-Perry Aggression Questionnaire; TSCRS, Teacher's Self-control Rating Scale; ABS, Scale of aggressive behavior; MMPI, Minnesota Multiphasic Personality Inventory; PFT, Rosenzweig picture frustration test items; JPI, Jackson Personality Inventory.

### 3.2 Characteristics of martial arts interventions and outcome measures

All included studies employed longitudinal intervention designs and involved children and adolescents ranging from primary school to high school levels. Sample sizes of individual studies ranged from 34 to 298 participants, with a total sample size of 1,352 across all studies. Most studies adopted quantitative non-randomized designs, with a smaller number employing randomized controlled trials.

In terms of intervention types, the martial arts disciplines included judo, karate, taekwondo, aikido, Chinese traditional martial arts, as well as modern or mixed martial arts training programs. Some studies implemented a single martial arts discipline, whereas others combined two or more martial arts forms within the intervention. The training orientations varied across studies, including traditional approaches emphasizing discipline and educational values, competitive or combat-oriented approaches, and mixed orientations integrating both elements.

The duration of interventions varied considerably across studies, ranging from 2.5 weeks to 2 years. Most interventions lasted between 10 weeks and 6 months. Training frequency typically ranged from one to three sessions per week, with session durations between 45 and 90 min. Some studies employed non-martial arts physical activities or regular physical education classes as control conditions, while others used wait-list controls or single-group pre–post designs. Detailed intervention parameters and study design characteristics are provided in Supplementary material A3.

Regarding outcome measures, all included studies examined aggressive behavior or related psychological and behavioral indicators as primary outcomes. Aggression outcomes included multiple dimensions, such as physical aggression, verbal aggression, indirect aggression, anger, and hostility. Most outcomes were assessed using validated standardized questionnaires. In addition to aggression outcomes, some studies also examined related psychological variables, including self-control, emotional management, self-esteem, and peer behavior. Further details are presented in Table 1.

With respect to gender, most studies did not conduct specific gender-stratified analyses. Only a few studies reported gender-related differences, and the existing evidence remains insufficient to establish a consistent pattern regarding potential moderating effects of gender.

### 3.3 Overall distribution of intervention effects

As shown in Table 2, the overall distribution of findings indicates that the relationship between martial arts interventions and aggressive behavior among children and adolescents does not follow a single consistent direction. Instead, the results demonstrate notable variation across martial arts disciplines and differences in the amount and consistency of available evidence.

**Table 2:** Effects of different martial arts forms on aggression in children and adolescents: a research-based classification.

Martial arts discipline	Number of studies	Direction of effects	Descriptive evidence label
Judo	6	Two reported an increase; two reported a decrease; two reported no change;	Inconsistent evidence
Karate	3	One reported a decrease; two reported no change;	Limited evidence
Taekwondo	2	One reported a decrease; one reported no change	Very limited evidence
Jiu-Jitsu	1	One reported no change	Very limited evidence
Chinese martial arts	1	One reported a decrease	Very limited evidence
Aikido	1	One reported no change	Very limited evidence
Modern martial arts	1	One reported an increase	Very limited evidence

The final column provides a descriptive evidence labels derived from the narrative synthesis rather than a formal certainty-of-evidence rating.

Labels were assigned based on two criteria: (1) the number of available studies for each martial arts discipline and (2) the consistency of the reported direction of effects across studies. “Inconsistent evidence” was used when multiple studies within the same discipline reported divergent directions of effect. “Limited evidence” was used when only a small number of studies were available and findings showed partial consistency. “Very limited evidence” was used when a discipline was represented by only one or two studies.

Among the martial arts disciplines with a relatively larger number of studies, judo showed substantial variation in outcomes. Some studies reported reductions in aggressive behavior, whereas others reported increases or no significant changes, indicating a high degree of heterogeneity in intervention effects. In comparison, the findings for karate and taekwondo appeared somewhat more consistent; however, the limited number of studies prevents firm conclusions regarding stable intervention trends.

In addition, several martial arts disciplines were represented by only a single study, including Chinese traditional martial arts, jiu-jitsu, aikido, and modern martial arts programs. In these cases, the observed changes in aggressive behavior should be considered exploratory, and the available evidence remains insufficient to support generalizable conclusions at the level of specific martial arts disciplines.

Overall, the current body of evidence is characterized more by within-discipline inconsistency and uneven distribution of studies across martial arts disciplines rather than by clearly differentiated intervention effects between martial arts styles. Consequently, most findings remain at the level of limited or very limited evidence.

### 3.4 Risk of bias

A total of 11 studies were included in this review, comprising two randomized controlled trials (S3 and S6) and nine non-randomized intervention studies. Among the randomized controlled trials, study S3 was judged to present some concerns regarding overall risk of bias, primarily related to the randomization process and outcome measurement. Study S6 was assessed as having a high risk of bias, mainly due to potential deviations from the intended interventions and limitations in the handling of participant attrition. For the non-randomized intervention studies, the overall methodological quality was considered moderate. Most studies clearly described the intervention procedures, measurement instruments, and statistical analysis methods. Outcome measures were generally assessed using standardized psychological scales, and the reliability of measurements and statistical procedures was generally appropriate.

However, several methodological limitations were observed across studies. First, some studies reported baseline differences between groups or did not fully report baseline comparability. Second, control of potential confounding factors was limited, with some studies not applying statistical adjustments for confounding variables. Third, reporting of follow-up completeness varied across studies, and a small number of studies showed relatively high attrition rates without adequate analysis of missing data. Finally, several earlier studies provided insufficient descriptions of group allocation procedures.

Overall, although the included studies generally demonstrated acceptable reporting of intervention procedures, outcome measurements, and statistical analyses, certain methodological limitations remain, particularly regarding baseline comparability and follow-up management. The distribution of risk-of-bias judgments across studies and methodological domains is summarized in the risk-of-bias figures provided in Supplementary material A1 (S2).

## 4 Discussion

### 4.1 Summary of direct evidence from included studies

Based on the 11 longitudinal intervention studies included in this review, the relationship between martial arts training and aggressive behavior among children and adolescents has not shown a stable or consistent directional pattern. Instead, the evidence demonstrates variation across martial arts disciplines and divergence across outcomes. Some studies reported reductions in aggression-related outcomes following martial arts interventions, particularly in the domain of physical aggression (39). However, other studies did not observe significant intervention effects, whereas others reported increases in aggression or hostility under specific training forms or contexts (40–42). Therefore, the available evidence is insufficient to support a general conclusion that martial arts training consistently reduces aggressive behavior in children and adolescents.

These mixed findings were observed both across different martial arts disciplines and within the same discipline. Among the martial arts forms examined, judo has been investigated in a relatively larger number of studies. However, intervention outcomes in judo studies showed clear divergence, including reports of reduced aggression (29, 39), as well as findings of no significant change or increases in aggression-related indicators (42–44). For karate, taekwondo, aikido, Chinese martial arts, and modern or mixed martial arts programs, the number of available studies remains limited and unevenly distributed across disciplines (14, 16, 40, 41). As a result, the current evidence is insufficient to make stable comparisons or rankings of intervention effects across different martial arts forms.

When considering different dimensions of aggressive behavior, physical aggression showed a decreasing trend in some studies (16, 39), but this pattern was not replicated across all martial arts programs. In contrast, outcomes related to verbal aggression, anger, hostility, relational aggression, bullying, and broader externalizing difficulties were more heterogeneous (14, 40, 42–44). Although these outcomes are all related to aggression, they are not conceptually equivalent, which limits direct comparison across studies. This suggests that martial arts interventions may be associated with aggression-related changes only under certain conditions, depending partly on how aggression is defined, measured, and situated within the intervention context.

At the level of direct evidence, these findings support a cautious interpretation: martial arts interventions were associated with mixed and context-dependent outcomes rather than a uniform protective effect. Accordingly, this review should not be interpreted as evidence that martial arts training generally reduces aggression among children and adolescents. Instead, it indicates the need to examine how intervention design, participant characteristics, outcome measurement, and cultural or educational context may shape the direction of reported findings. The following sections further discuss these sources of heterogeneity, as well as the psychological variables reported in the included studies that may help explain the mixed findings.

### 4.2 Potential sources of heterogeneity across studies

The heterogeneity observed in the included studies may be related to several review-level factors. The studies differed substantially in research design. Only two included studies used randomized controlled designs, whereas most adopted quasi-experimental or other non-randomized intervention designs. Differences in group allocation, baseline comparability, control conditions, attrition handling, and control of confounding factors may have influenced the direction and strength of intervention effects. Therefore, the inconsistency across studies may not be attributable solely to martial arts training itself, but may also reflect methodological differences. These design-related limitations reduce confidence in drawing strong causal conclusions from the current evidence base.

The intervention characteristics also varied considerably across studies. The included studies differed in martial arts discipline, intervention duration, training frequency, session length, instructional setting, and pedagogical orientation. Further comparison of training forms and instructional orientations suggests that the intervention outcomes did not show a clear distinction between “traditional/educational” and “competitive/combative” orientations. Some programs emphasizing discipline, philosophical education, and self-restraint reported reductions in aggression or anger in individual studies, such as Chinese traditional martial arts or education-oriented martial arts curricula (16), although this finding should be interpreted cautiously because the study was judged to have a high overall risk of bias. However, other education-oriented or school-based programs did not observe significant improvements in aggression-related outcomes (14). Similarly, training forms involving stronger combative or competitive elements were associated with increases in aggression or hostility in some studies (40), whereas other martial arts programs with similar training orientations did not show comparable increases, instead reporting no significant changes in anger or aggression-related outcomes (44, 45). Given that most martial arts forms were represented by only one or a small number of studies, and that findings were inconsistent even within similar training orientations, the current evidence is insufficient to support a stable classification of intervention effects based solely on a “traditional–competitive” framework.

Heterogeneity may also have arisen from differences in the operationalization of outcome variables. Although all included studies reported aggression-related outcomes, they did not measure the same construct in the same way. Some studies focused on specific dimensions of aggression, whereas others assessed anger, hostility, bullying-related behavior, delinquency-related or problem behavior indicators, or broader externalizing difficulties. In addition, the measurement tools differed across studies, which may have affected each study's sensitivity to detecting intervention-related changes. Therefore, the inconsistency of findings may partly reflect differences in how aggression-related outcomes were operationalized, rather than differences in intervention effectiveness alone.

Participant characteristics may also represent an important source of heterogeneity. The included studies differed in age range, gender composition, risk status, and school context. However, these factors were rarely examined formally as moderators, and most studies did not provide sufficient subgroup analyses to determine how participant characteristics shaped intervention effects. Among these factors, gender may be a moderator worthy of attention, although the current evidence remains limited. Only a small number of included studies specifically examined gender differences, and some findings suggested that the intervention effects of martial arts training may be more evident among boys (46). However, because the number of relevant studies is limited, the current evidence is insufficient to draw clear conclusions regarding gender-specific patterns. Future studies should more systematically examine whether gender and other individual characteristics moderate the effects of martial arts interventions on aggressive behavior.

Taken together, the heterogeneity observed in this review may reflect the combined influence of research design, intervention structure, instructional orientation, outcome measurement, and participant characteristics. These sources of heterogeneity, together with the limited number of randomized trials and the uneven distribution of studies across martial arts disciplines, lower confidence in any broad conclusion regarding the effects of martial arts training on aggression-related outcomes. In addition, the existing evidence suggests that changes in aggression-related outcomes were often reported or interpreted alongside psychological variables such as self-control, emotion regulation, self-esteem, or social adaptation. However, these psychological indicators were conceptualized and measured differently across studies. This pattern suggests that psychological variables may provide useful interpretive dimensions for understanding the mixed findings, but the current evidence does not establish them as confirmed mechanisms of change. The following section therefore discusses the psychological variables directly measured or reported in the included studies.

### 4.3 Psychological variables directly measured or reported in included studies

The included studies show that the relationship between martial arts training and changes in aggressive behavior is often reported or interpreted alongside several psychological variables. These variables include self-control (41), anger expression and anger control (44), emotional management or emotional intelligence (29, 39), executive functions and self-esteem (45), self-concept and motivational climate (29), and peer-related or psychosocial indicators such as helpful bystander behavior, victimization, emotional difficulties, and behavioral difficulties (14, 46). Although these variables were not measured using a unified framework, they were narratively grouped into two broad domains for synthesis: self-regulation-related variables, including self-control, anger-related indicators, emotional management, and executive functions; and socialization-related variables, including self-esteem, self-concept, motivational climate, helpful bystander behavior, victimization, and broader psychosocial functioning. The following sections discuss these two domains as organizing categories based on the psychological variables represented in the included studies, while explicitly distinguishing direct evidence from broader theoretical interpretation.

For interpretive clarity, the following discussion distinguishes among three levels of inference. The first level, empirically grounded findings, refers to psychological variables that were directly measured or reported in the included studies. The second level, review-level interpretations, refers to recurring patterns observed across studies, such as the clustering of variables around self-regulation and socialization domains. The third level, theoretical explanations, draws on broader developmental and social psychological literature, including concepts such as executive function, emotion regulation, and norm internalization, to contextualize these patterns. Importantly, these theoretical explanations provide plausible interpretive routes but remain inferential in nature and require confirmatory testing in future empirical research.

#### 4.3.1 Self-control and emotion regulation measures

At the level of empirically grounded findings, self-regulation-related variables were among the most commonly reported psychological constructs in the included studies. These variables were mainly represented by self-control, emotion-related indicators, anger expression, anger control, and executive functions. However, the findings were not consistent across studies. Some school-based martial arts or judo interventions reported improvements in emotional or psychosocial indicators alongside reductions in aggression-related outcomes, whereas other studies examining self-control, anger, or executive functions did not observe clear or consistent improvements. In particular, evidence concerning executive functions remains limited and is mainly derived from a small number of studies. Therefore, although self-regulation-related variables are valuable for interpreting the relationship between martial arts training and aggression, the current evidence supports their relevance as reported psychological variables but is insufficient to confirm self-regulation as an established mechanism of change.

Beyond the direct evidence from the included studies, broader developmental psychology literature provides a theoretical rationale for why self-regulation may be relevant to aggression-related outcomes. Previous studies have shown that deficits in executive functions, particularly inhibitory control and cognitive flexibility, are closely associated with impulsive aggression among adolescents (47, 48). When individuals have difficulty inhibiting impulsive responses or integrating contextual information during conflict situations, they may be more likely to develop hostile attribution biases and display aggressive behavioral responses (49, 50). Within martial arts contexts, related studies have also suggested that training environments emphasizing rule adherence, self-restraint, and controlled movement may provide practical opportunities for practicing and strengthening self-control (51, 52).

Emotion regulation also provides a plausible theoretical perspective for interpreting aggression-related outcomes. Impaired emotion regulation is considered one of the important risk factors for behavioral dysregulation among adolescents, making individuals more likely to respond impulsively or aggressively under conditions of heightened emotional arousal. In the broader intervention literature, physical activity is generally considered to improve adolescents' emotion regulation capacity in stressful situations to some extent (53). More specifically within martial arts training contexts, some studies have observed improvements in anger management or emotional control among participants (54). At the same time, martial arts teaching models that emphasize martial morality and rule awareness may provide a supportive context for emotional regulation by enhancing emotional awareness and impulse monitoring (55). However, similar to aggression-related outcomes, changes in self-control and emotion-related variables did not show a consistent direction across all studies. Moreover, most included studies did not formally examine whether self-control, emotion regulation, or anger control mediated the relationship between martial arts training and aggression-related outcomes.

Overall, self-control and emotion regulation provide an important psychological perspective for understanding the divergent findings regarding martial arts training and aggressive behavior. However, the current evidence is more consistent with a context-dependent interpretation: different training structures and instructional orientations may shape how these variables are expressed, rather than producing a stable effect in a single direction. Accordingly, self-control, emotion regulation, and executive functions should be interpreted as theoretically plausible explanatory dimensions rather than confirmed mediating mechanisms. Claims about these variables as mechanisms linking martial arts training to aggression-related outcomes should therefore be treated as hypothesis-generating rather than confirmatory.

#### 4.3.2 Social adaptation and self-concept measures

At the level of empirically grounded findings, several included studies also reported psychological variables related to social adaptation and self-concept. These variables were represented in different forms, including self-esteem, self-concept, peer-related behavior, attitudes toward aggression, victimization-related indicators, and broader psychosocial functioning. Some studies examined whether martial arts training was accompanied by changes in self-esteem, self-concept, or peer behavior, whereas others included attitudes toward aggression, victimization, or broader social adaptation as part of the intervention outcomes. However, similar to self-regulation-related variables, the findings were not fully consistent across studies, and these variables differed in measurement instruments and theoretical positioning. Therefore, the included studies suggest that the relationship between martial arts training and aggression-related outcomes can be understood not only from the perspective of individual self-regulation, but also from the perspective of social development. At the same time, the current evidence does not provide strong support for social adaptation or self-concept as confirmed causal pathways linking martial arts training to aggression-related outcomes.

Beyond the direct evidence from the included studies, broader social-developmental theory provides a theoretical rationale for why socialization-related variables may be relevant to aggression. Social norm internalization refers to the process through which individuals gradually transform external behavioral rules, moral standards, and social expectations into stable behavioral standards, and it represents an important psychological dimension of adolescent socialization (56). This process is usually embedded in peer interaction and social feedback, through which acceptance, reinforcement, or rejection shapes individuals' understanding of behavioral consequences and social values (57). Among adolescents, peer acceptance is closely related to the development of both aggressive and prosocial behavior. Its role may not lie in directly suppressing aggression, but rather in indirectly shaping behavioral choices through normative expectations and value judgments (58).

Martial arts training may provide a relatively structured social context for this process. Traditional or education-oriented martial arts practices often emphasize etiquette, respect for instructors, and non-violent values, and may reinforce the behavioral meaning of these norms through repeated training situations. For example, one program that combined martial arts training with philosophical and moral education reported more favorable aggression-related outcomes (16). Related literature also suggests that the social climate and pedagogical orientation of martial arts training may shape whether participants develop prosocial experiences or reinforce competitive and dominance-oriented meanings (59). In this sense, repeated cycles of rules, feedback, and identification in long-term training may help external norms become more internalized standards of behavior (60), supporting a gradual shift from external regulation toward self-regulation (61).

However, this interpretation also requires caution. Not all martial arts or combat-based training environments naturally promote social adaptation or norm internalization. In contexts where competitive achievement, physical dominance, or confrontation is emphasized as the primary goal, and where clear value guidance is lacking, peer interaction may reinforce performance-oriented or dominance-related norms rather than prosocial norms. Therefore, the socialization-related effects of martial arts training may depend strongly on instructional orientation, group climate, teacher values, and how discipline and respect are enacted during practice.

Overall, social adaptation-related variables, including peer acceptance, norm identification, and self-concept, provide an important socialization perspective for understanding the heterogeneous relationship between martial arts training and aggression. These variables may not operate by directly suppressing aggression, but rather by shaping individuals' understanding of social rules, behavioral consequences, and moral values, thereby indirectly influencing behavioral choices in conflict situations. This process may interact with self-control and emotion regulation as part of a broader psychological framework for interpreting martial arts interventions targeting aggression. Nevertheless, because most included studies did not directly test mediation or moderation involving social adaptation-related variables, these variables should be understood as plausible explanatory dimensions represented in the existing research, rather than as confirmed mechanisms of change.

### 4.4 Cultural and educational contexts of martial arts interventions

The heterogeneity observed across the included studies may also be related to differences in the cultural and educational contexts in which martial arts interventions were implemented. Martial arts should not be understood simply as culturally neutral or homogeneous forms of physical activity. Rather, the psychological and behavioral meanings of martial arts training may depend on how it is positioned, for example, as traditional practice, school physical education, competitive sport, self-defense training, moral education, behavioral intervention, or general youth development activity. Previous reviews have also noted that the social-psychological outcomes of martial arts participation are not determined by technical style alone, but may also be influenced by martial arts type, participant characteristics, training orientation, and social background (22, 62).

Across the included studies, martial arts interventions conducted in different countries and regions differed not only in geographical setting, but more importantly in how they were positioned within educational and social systems. Some programs framed martial arts as traditional practice integrating philosophical education, moral instruction, and behavioral norm training. For example, one Chinese martial arts program conducted in Hong Kong/China explicitly incorporated philosophical and moral education elements and reported reductions in both reactive and proactive aggression (16). Other studies embedded martial arts within school physical education or extracurricular activity settings, with greater emphasis on physical activity, emotional development, and school adaptation. This pattern was evident in several Spanish studies on judo or karate, which reported mixed findings across aggression-related, emotional, and psychosocial outcomes (29, 39, 44). In addition, some studies positioned martial arts as school violence prevention, youth behavioral intervention, long-term sport participation, or training for at-risk youth. These different framings may influence how participants understand discipline, confrontation, respect, and self-control (14, 40–43, 45, 46).

These differences suggest that the effects of martial arts training may depend not only on the techniques being taught, but also on the meanings attached to training. A program emphasizing etiquette, respect, self-restraint, non-violence, and moral reflection may constitute a very different developmental context from one organized primarily around competitive performance, physical dominance, or self-defense. In this sense, cultural and educational frameworks may shape how adolescents understand discipline, confrontation, authority, emotional restraint, peer relationships, and the appropriate use of physical force. Similar technical activities may therefore carry different psychological meanings across different contexts.

However, cultural origin itself should not be understood as determining intervention effects. The current evidence does not support a simple distinction between “Eastern–Western” contexts or between “traditional–competitive” martial arts. The more important issue is how martial arts are taught, what values instructors emphasize, how discipline and authority are managed, and how peer interaction is organized within the intervention. This point is particularly important because even within the same martial arts discipline, such as judo, different studies reported inconsistent findings. Therefore, the cultural analysis in this review should be understood as an examination of pedagogical meaning and social framing, rather than as a fixed comparison between countries or martial arts origins.

The transferability of the current findings should therefore be considered limited and conditional. Findings from structured school-based or youth development programs may be most relevant to settings in which martial arts training is delivered with explicit educational goals, rule-based instruction, non-violence norms, and instructor-guided reflection. In contrast, findings from programs involving at-risk youth, short-term behavioral interventions, highly specific cultural or institutional contexts, or narrowly defined training goals should not be assumed to generalize directly to ordinary school physical education, community martial arts clubs, competitive training environments, or self-defense-oriented programs. At present, the evidence does not support broad generalization across educational, cultural, or social settings without careful consideration of program goals, instructor values, participant characteristics, peer climate, and the way aggression and discipline are addressed during training.

Future studies should therefore report the cultural and educational contexts of martial arts interventions more explicitly. In addition to describing the martial arts discipline, intervention dose, and participant characteristics, researchers should specify whether the program is positioned as moral education, competitive training, self-defense, school physical education, behavioral intervention, or health promotion. They should also report the values emphasized by instructors, the role of etiquette and discipline, the nature of peer interaction, and how non-violence and aggression are addressed during training. Only with more detailed reporting of these elements will future reviews be able to examine cultural and pedagogical moderators more systematically and further explain why martial arts interventions produce heterogeneous aggression-related outcomes.

### 4.5 Implications for future research and practice

The findings of this review have several implications for future research and practice in sport and exercise psychology. First, martial arts training should be specified more precisely rather than treated as a single intervention category when examining its psychological or behavioral outcomes. Future studies should describe not only the martial arts discipline and intervention dose, but also the instructional orientation, training structure, competitive emphasis, pedagogical environment, social interaction patterns, and value frameworks embedded within the program. Such information is necessary for understanding why similar martial arts forms may produce different aggression-related outcomes across studies.

Second, the present synthesis indicates that psychological variables associated with self-regulation and socialization may provide a useful analytical lens for understanding the relationship between martial arts training and aggressive behavior. However, these variables should be incorporated more explicitly into future study designs. Rather than measuring psychological variables only as secondary outcomes, future research should examine whether constructs such as self-control, emotion regulation, peer interaction, self-concept, and normative awareness function as mediators, moderators, or parallel developmental outcomes. Methodologically, this will require stronger longitudinal or randomized designs, clearly specified comparison conditions, repeated measurements, and sufficient reporting of intervention processes. This would help clarify not only whether martial arts interventions are effective, but also under what psychological conditions and through which processes they may influence aggression-related outcomes.

Third, future research should pay greater attention to cultural and educational context. Martial arts interventions may carry different meanings depending on whether they are framed as traditional practice, school physical education, competitive sport, self-defense training, moral education, behavioral intervention, or general youth development activity. Researchers should therefore report how programs are presented to participants, what values instructors emphasize, and how discipline, respect, non-violence, and aggression are addressed during training. Such reporting would allow future reviews to examine cultural and pedagogical moderators more systematically.

From a practical perspective, the findings suggest that martial arts training should not be assumed to reduce aggression simply by increasing physical activity or exposure to combat-related skills. Programs intended to reduce aggression or support youth development should explicitly integrate rule awareness, emotional regulation, respect for others, non-violent conflict resolution, and positive peer interaction into the training process. Instructors and schools should also be attentive to the possibility that competitive or dominance-oriented climates may weaken the developmental value of martial arts training if not balanced by clear educational guidance.

Overall, martial arts training may provide a useful context for examining broader questions in sport and exercise psychology concerning structured physical activity, behavioral regulation, and social development. However, future studies should move beyond simple intervention evaluations and adopt theoretically informed, culturally sensitive, and methodologically rigorous designs that clarify when, for whom, and through what processes martial arts training may influence aggression-related outcomes in children and adolescents.

## 5 Limitations

Although this review systematically synthesized longitudinal evidence on martial arts interventions targeting aggressive behavior in children and adolescents, several limitations should be considered when interpreting the findings.

First, the evidence base was limited in size and methodological robustness. Only 11 longitudinal intervention studies were included, and most martial arts disciplines were represented by one or a small number of studies. In addition, most included studies used quasi-experimental or pre–post designs rather than randomized controlled designs, which limits causal inference and increases the possibility that baseline differences, uncontrolled confounding, attrition, or contextual factors may have influenced the observed effects. The included studies also differed substantially in participant characteristics, intervention duration, training orientation, comparison conditions, and outcome measures. These design-related and intervention-related differences reduce confidence in drawing strong causal or comparative conclusions about the effects of martial arts training on aggression-related outcomes. Therefore, the findings should be interpreted as a cautious synthesis of emerging and heterogeneous evidence rather than as generalizable conclusions about martial arts training as a whole.

Second, limitations also relate to the scope and retrieval of evidence sources. This review mainly included peer-reviewed journal articles published in English or Chinese and did not systematically incorporate gray literature. The PROSPERO registration was retrospective and therefore should be interpreted as a transparency measure rather than as prospective protocol registration. In addition, the database search was conducted up to January 2025, and specialist databases such as PsycINFO and SPORTDiscus were not searched. Finally, 172 records could not be retrieved in full text despite multiple retrieval attempts. Together, these factors may have limited the comprehensiveness and representativeness of the final evidence base and may have increased the risk of retrieval, language, or publication-related bias.

Third, the psychological variables synthesized in this review were heterogeneous in definition, measurement, and theoretical framing. Although variables such as self-control, emotion regulation, self-concept, and social adaptation were identified across the included studies, most studies did not formally test mediation, moderation, or causal pathways. As a result, the psychological-process categories proposed in this review should be understood as functional syntheses of reported variables rather than as direct evidence of confirmed mechanisms. This limits the extent to which the review can explain why or under what psychological conditions martial arts interventions may influence aggression-related outcomes.

Finally, most included studies provided limited information on cultural framing, instructor values, pedagogical orientation, and the way discipline, respect, competition, or non-violence were enacted during training. This restricted the extent to which cultural and educational context could be analyzed systematically as a potential moderator of intervention effects. These contextual reporting limitations also constrain the transferability of the findings across different educational systems, cultural settings, community environments, and martial arts delivery models. Future research should provide more detailed reporting of intervention content, psychological variables, cultural context, and study design to support more precise synthesis and stronger causal inferences.

## 6 Conclusion

This review synthesized evidence from 11 longitudinal intervention studies examining the relationship between martial arts training and aggressive behavior among children and adolescents. The available evidence does not indicate a consistent directional pattern of intervention effects. Instead, changes in aggressive behavior varied across different martial arts disciplines, training orientations, and intervention contexts, and even within the same discipline the reported outcomes lacked consistent replication. These findings suggest that treating martial arts training as a homogeneous intervention may not adequately capture the complexity observed in existing research.

At the level of psychological variables, the included studies reported constructs related to self-control, emotion regulation, self-concept, and broader socialization-related factors. However, substantial variation existed in how these variables were conceptualized and measured, and most studies did not formally test mediation, moderation, or causal pathways. These findings indicate that psychological variables are relevant to this literature, but their explanatory role remains to be tested more directly in future research.

Overall, the present review suggests that martial arts training should not be interpreted as having a uniform aggression-reducing effect among children and adolescents. Rather, it may serve as a potentially useful developmental context under certain conditions, particularly when training is delivered with clear educational goals, rule-based instruction, non-violence norms, and supportive pedagogical guidance. Future research should employ more rigorous study designs, larger sample sizes, clearer intervention reporting, and more consistent frameworks for measuring psychological variables. Such efforts may help identify when, for whom, and through what processes martial arts interventions may contribute to aggression-related outcomes and youth development.
