# Supplementary material for: Martial arts training and aggressive behavior in children and adolescents: a systematic review of longitudinal evidence and psychological processes
Source: Front Public Health. 2026 Jul 17;14:1838396. doi: 10.3389/fpubh.2026.1838396 (PMC13423899; doi:10.3389/fpubh.2026.1838396)
Supplement: Supplementary file 2 [file Table_2.docx]

**Codebook for Data Extraction**

This codebook describes the variables extracted from each included study for the systematic review on martial arts training and aggressive behaviour.

**Study ID**

Unique identifier assigned to each included study (e.g., S1, S2), used to ensure consistency across tables, figures, and supplementary materials.

**Title**

Full title of the original study as published.

**Author(s) and year**

Surname of the first author followed by year of publication.

**Country / Region**

Country or region where the study was conducted.

**Study Design**

Study design of each included study as reported in the original publication (e.g., randomized controlled trial or non-randomized intervention study). Randomized controlled trials were assessed using the Cochrane Risk of Bias 2 (RoB 2) tool, while non-randomized intervention studies were assessed using the Joanna Briggs Institute (JBI) Critical Appraisal Checklist.

**Participants**

Description of the study population, such as children, adolescents, or students.

**Sample Size** **(N)**

Total number of participants included in the study analysis.

**Age/Grade**

Mean age and standard deviation, age range, or school grade of participants, as reported in the original study.

**Gender_Male (%)**

Percentage of male participants in the sample. If not reported, coded as “NR”.

**Intervention (Martial Arts Discipline)**

Specific martial art(s) examined, such as traditional Chinese martial arts, Taekwondo, Karate, Judo, Aikido, MMA, or unspecified martial arts.

**Training Orientation**

Primary orientation of the martial arts practice (e.g., traditional, competitive, educational, recreational, or mixed), classified based on descriptions provided in the original studies.

**Intervention Duration**

Duration of the martial arts training program (e.g., total intervention period in weeks or months).

**Intervention Frequency and Session Duration**

Training frequency (sessions per week) and duration of each training session (minutes) in the intervention program.

**Comparison / Control Condition**

Description of control or comparison groups, such as non-martial arts participants, other sports groups, or no-exercise controls. If no comparison group was used, coded as “None”.

**Measurement Instruments**

Names of standardized questionnaires or assessment tools used to measure aggression or related psychological variables.

**Primary Outcome Variables**

Primary outcome variables assessed in each study, including aggression-related outcomes (e.g., aggression, hostility, anger, violence, bullying behaviour) and related psychological variables reported alongside aggression outcomes (e.g., self-control, emotion regulation, self-esteem, peer relations, or social adaptation).

**Main Finding Direction**

Overall direction of the association or effect of martial arts participation on aggression-related outcomes, coded as decrease, increase, mixed, or no change.

**Key Findings Summary**

Brief qualitative summary of the main results reported by the authors.

**Gender-related Analysis**

Indicates whether sex or gender differences were examined or discussed in the study.

**Notes**

Additional comments on methodological features, limitations, missing information, or specific issues relevant to interpretation.
