# Supplementary material for: Martial arts training and aggressive behavior in children and adolescents: a systematic review of longitudinal evidence and psychological processes
Source: Front Public Health. 2026 Jul 17;14:1838396. doi: 10.3389/fpubh.2026.1838396 (PMC13423899; doi:10.3389/fpubh.2026.1838396)
Supplement: Supplementary file 3 [file Table_3.docx]

# **S1. Literature search strategy**

## **PubMed**

**#1**

((((((adolescents[Title/Abstract]) OR (youth[Title/Abstract])) OR (students[Title/Abstract])) OR (teenager[Title/Abstract])) OR (children[Title/Abstract])) OR (boys[Title/Abstract])) OR (girls[Title/Abstract])

**#2**

((((((((((martial arts[Title/Abstract]) OR (kung fu[Title/Abstract])) OR (wushu[Title/Abstract])) OR (Tai Chi[Title/Abstract])) OR (taiji[Title/Abstract])) OR (Taekwondo[Title/Abstract])) OR (Karate[Title/Abstract])) OR (Judo[Title/Abstract])) OR (Aikido[Title/Abstract])) OR (MMA[Title/Abstract])) OR (mixed martial arts[Title/Abstract])

**#3**

(((((((aggression[Title/Abstract]) OR (aggressive[Title/Abstract])) OR (aggressiveness[Title/Abstract])) OR (hostility[Title/Abstract])) OR (violence[Title/Abstract])) OR (anger[Title/Abstract])) OR (bully[Title/Abstract])) OR (bullying[Title/Abstract])

**#4**

#1 AND #2 AND #3

## **Web of Science**

**#1**

((((((TS=(adolescents)) OR TS=(youth)) OR TS=(students)) OR TS=(teenager)) OR TS=(children)) OR TS=(boys)) OR TS=(girls)

**#2**

((((((((((TS=(martial arts)) OR TS=(kung fu)) OR TS=(wushu)) OR TS=(Tai Chi)) OR TS=(taiji)) OR TS=(Taekwondo)) OR TS=(Karate)) OR TS=(Judo)) OR TS=(Aikido)) OR TS=(MMA)) OR TS=(mixed martial arts)

**#3**

(((((((TS=(aggression)) OR TS=(aggressive)) OR TS=(aggressiveness)) OR TS=(hostility)) OR TS=(violence)) OR TS=(anger)) OR TS=(bully)) OR TS=(bullying)

**#4**

#1 AND #2 AND #3

## **ScienceDirect**

**Title, abstract or author-specified keywords：**

("martial arts" OR "Taekwondo" OR "Karate" OR "Judo" OR "Aikido") AND ("aggressive" OR "hostility")

**Title：**

("school bullying" OR "aggression" OR "hostility") AND ("martial arts" OR "mixed martial arts")

## **Chinese databases, including China National Knowledge Infrastructure (CNKI) and Wanfang Data, were also searched using the same search strategy.**

**#1**

(青少年 OR 儿童 OR 学生 OR 少年)

**#2**

(武术 OR 武术运动 OR 传统武术 OR 中国武术 OR 散打 OR 太极 OR 跆拳道 OR 空手道 OR 柔道)

**#3**

(攻击 OR 攻击性 OR 攻击行为 OR 校园欺凌 OR 校园霸凌 )

**#4**

#1 AND #2 AND #3

The final literature search across all databases was conducted on 23 January 2025.

# **S2. Risk-of-bias assessment of included studies (RoB 2 and JBI tools)**


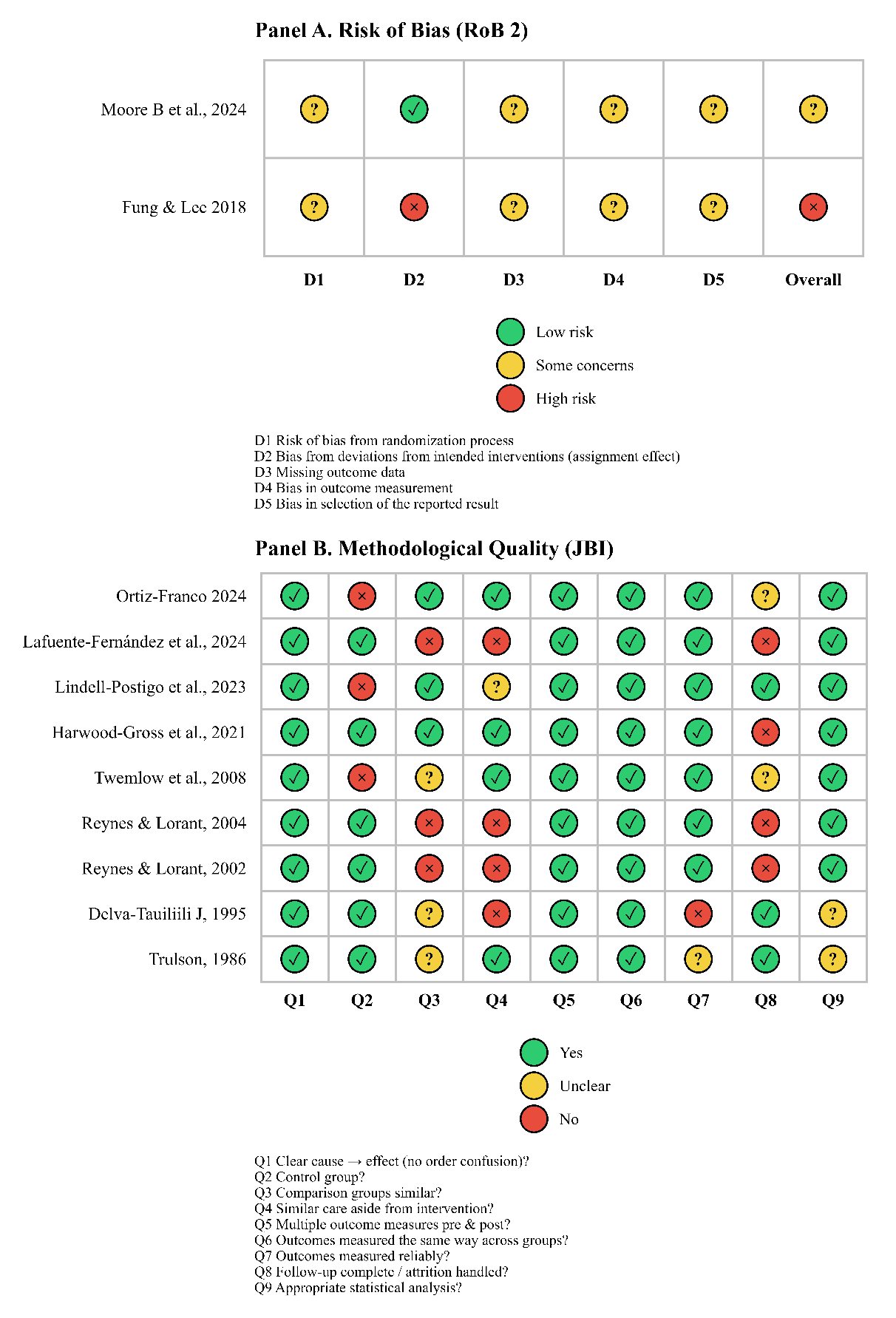


Fig. 1 Traffic-light plot showing the risk-of-bias ratings for each included study assessed using the RoB 2 tool for randomized controlled trials and the JBI critical appraisal checklist for quasi-experimental studies


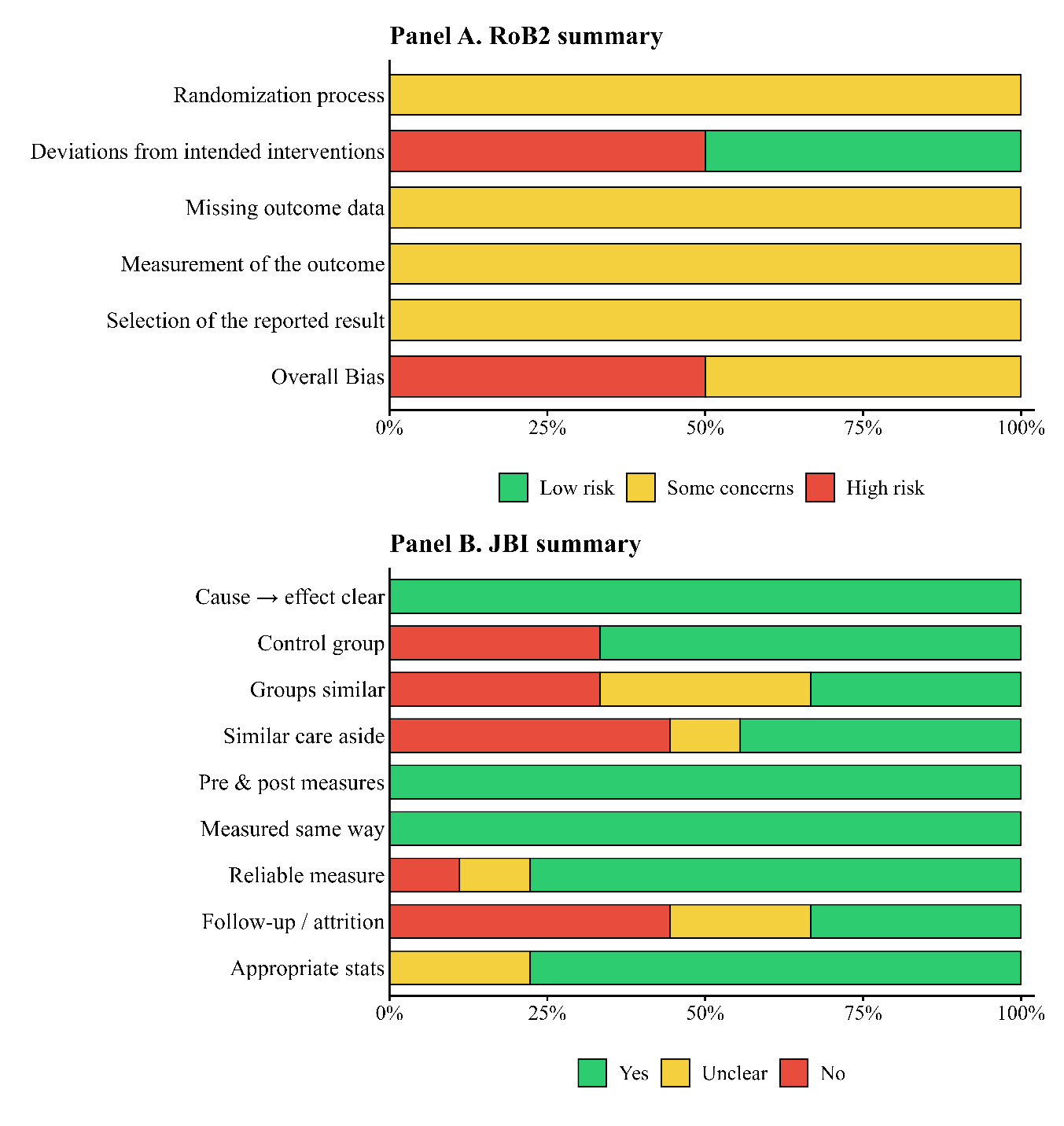


Fig. 2 Summary plot showing the distribution of risk-of-bias judgments across methodological domains.
